# Supplementary material for: Dynamic monitoring of viral gene expression reveals rapid antiviral effects of CD8 T cells recognizing the HCMV-pp65 antigen
Source: Front Immunol. 2024 Jul 15;15:1439184. doi: 10.3389/fimmu.2024.1439184 (PMC11299495; doi:10.3389/fimmu.2024.1439184)
Supplement: Supplementary file 1 [file DataSheet_1.pdf]

Supplementary Figures

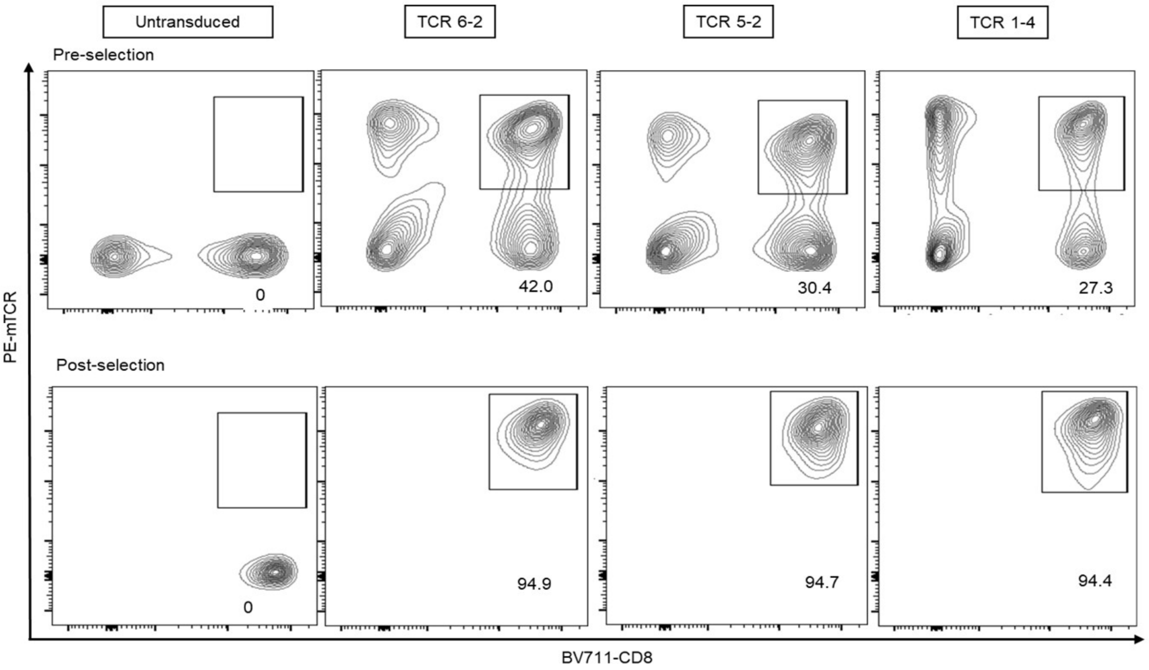

Supplementary Figure 1

Flow cytometric validation of transgenic CD8+ mTCR+ cell purification efficiency. CD8 T cells were transfected with retroviruses expressing the indicated TCR and grown for 3-7 days. Transduction efficiency was validated by Flow cytometry staining of CD8+ mTCR+ cells (upper panel) and the same was performed upon MACS-sorting of mTCR+ CD8 T cells (lower panel) to control the purity of T-cells used in co-culture experiments. Representative purities are shown, no cultures with <80% of mTCR+ cells have been used in any experiment.

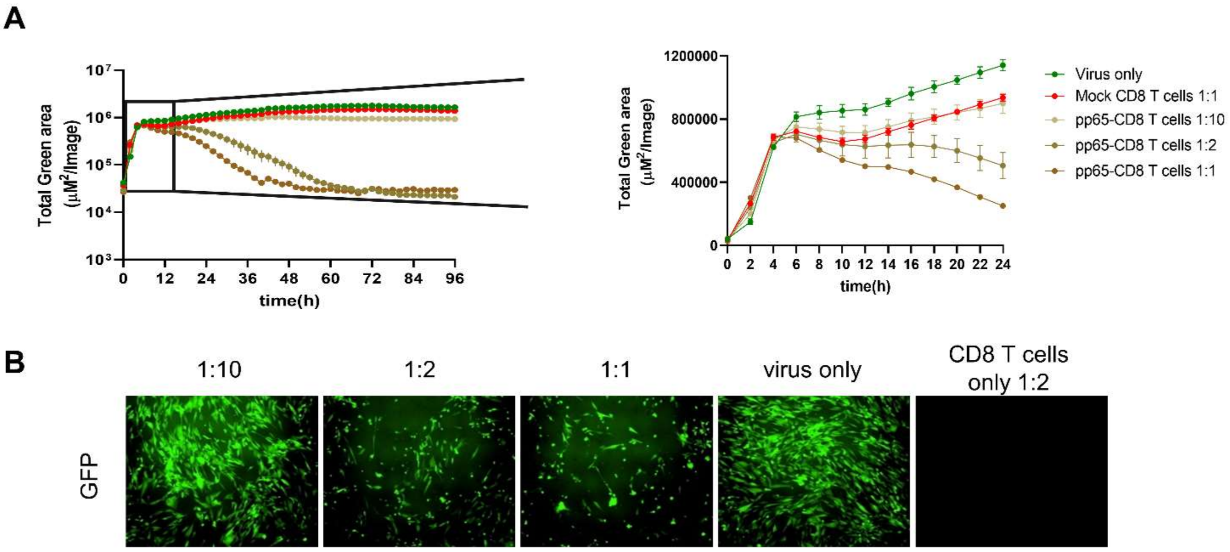

Supplementary Figure 2

Early expression of pp65 gene and recognition by pp65-specific CD8 T cells. MRC-5 cells were infected with TB40^{IEmNG} at a MOI of 1 and co-cultured with pp65-specific CD8 T cells transfected with the Intermediate-avidity TCR 1-4 at 1:10, 1:2 and 1:1 E:T. Total Green area indicated on the y-axes corresponds to mNeonGreen-ie1/2 expression, indicating viral loads plotted for up to 96h (Left panel) or 24 hours (Right panel) post infection in ARMATA. Each data point corresponds to the average ± SEM of biological duplicates from two independent experiments. (B) Representative images of co-culture at 24 hpi showing control of TB40^{IEmNG}- infected MRC-5 cells at indicated E:T ratios of Intermediate-avidity CD8 T cells.

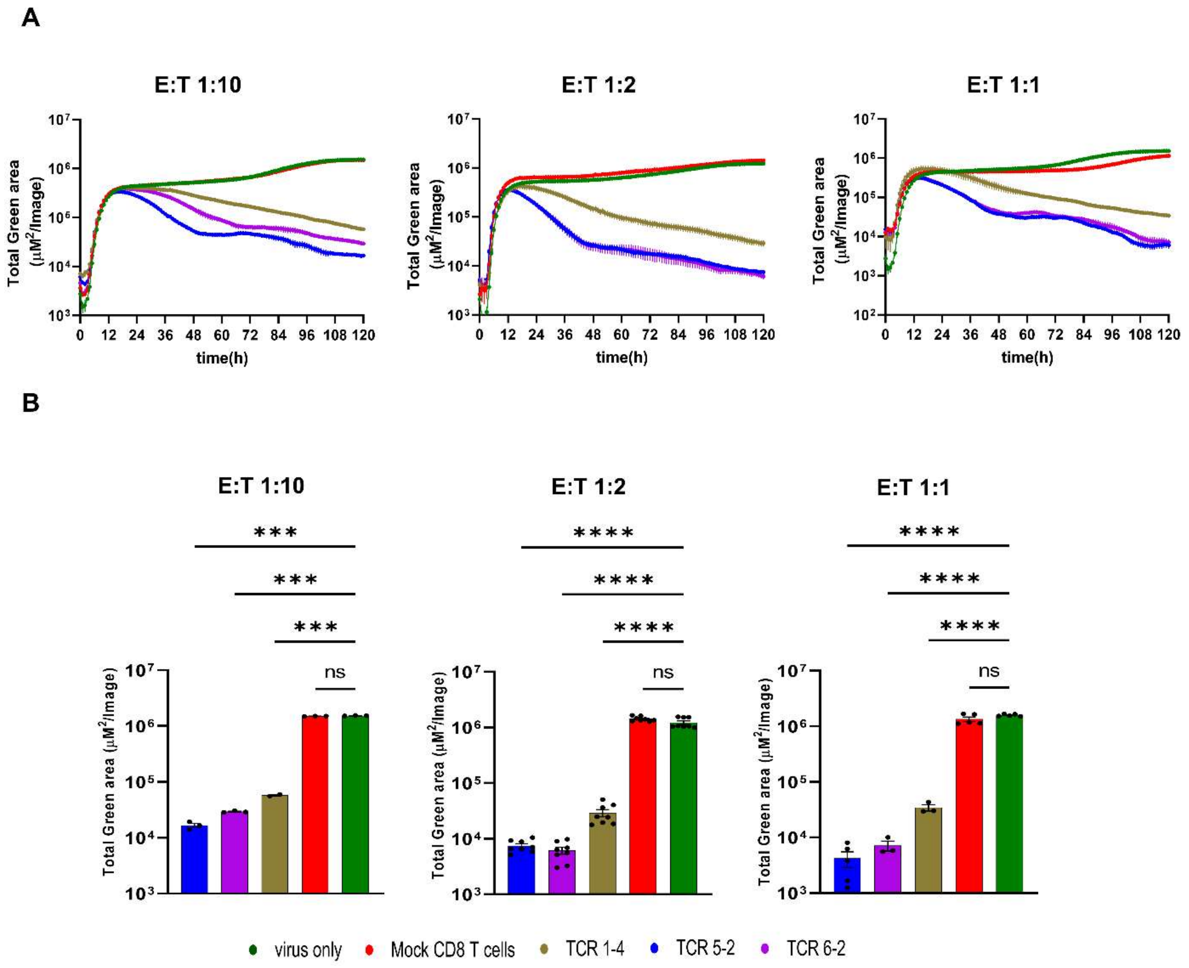

Supplementary Figure 3

Antiviral activity of transgenic pp65-specific CD8 T cells against $HCMV^{3F}$. (A) MRC-5 cells were infected with $TB40/HCMV^{3F}$ at MOI of 0.1 and co-cultured with CD8 T cells transfected with indicated pp65-specific TCRs at 1:10, 1:2 and 1:1 E:T. Total Green area indicated on the y-axes corresponds to mNeonGreen-ie1/2 expression indicating viral loads plotted for up to 120 hpi in ARMATA. (B) Representative plots showing endpoint green area. Each symbol corresponds to a biological replicate at indicated E:T ratios, pooled from ≥3 independent experiments. Histograms depict means ± SEM. Statistical analysis was done using Welch ANOVA and Dunnett's T3 test. ***$P < 0.001$ ****$P < 0.0001$, $P > 0.05$ not significant (ns).

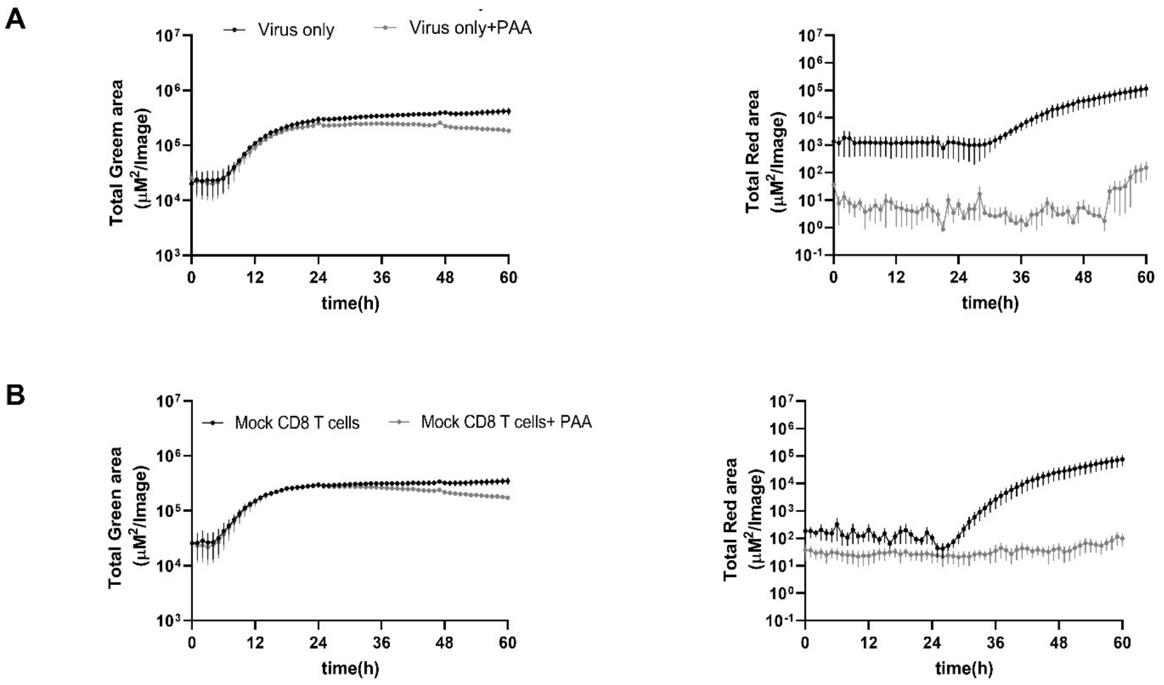

Supplementary Figure 4

Immediate-early and late gene expression dynamics of HCMV^{3F} reporter genes in the presence or absence of PAA. MRC-5 cells were infected with HCMV^{3F} at MOI 0.1 and were either left untreated or treated with PAA (100 µg/ml) at the time of infection. Total Green area shown on y-axes of graphs on the left side corresponds to mNeonGreen-ie1/2 while Total Red area shown on y-axes of graphs on the right side represent SCP-mCherry expression, indicating viral loads in distinct stages of the lytic replication cycle plotted for up to 60 hpi. Panel A shows signals in cells infected in absence of any T cells, and panel B the cells that were treated with mock-TCR transfected CD8 T cells. Each data point corresponds to independent biological replicates (n=4) from two individual experiments. Data with error bars depict mean ± SEM.
